# Associations Between the Perceived Severity of the COVID-19 Pandemic, Cyberchondria, Depression, Anxiety, Stress, and Lockdown Experience: Cross-sectional Survey Study

**DOI:** 10.2196/31052

**Published:** 2021-09-16

**Authors:** Lei Han, Yanru Zhan, Weizi Li, Yuqing Xu, Yan Xu, Jinzhe Zhao

**Affiliations:** 1 School of Psychology Shandong Normal University Ji’nan, Shandong China; 2 Faculty of Psychology Beijing Normal University Beijing China

**Keywords:** COVID-19, cyberchondria, depression, anxiety, stress, ABC theory of emotions, lockdown experience, perceived severity, cross-sectional, online health information

## Abstract

**Background:**

The outbreak of the COVID-19 pandemic has caused great panic among the public, with many people suffering from adverse stress reactions. To control the spread of the pandemic, governments in many countries have imposed lockdown policies. In this unique pandemic context, people can obtain information about pandemic dynamics on the internet. However, searching for health-related information on the internet frequently increases the possibility of individuals being troubled by the information that they find, and consequently, experiencing symptoms of cyberchondria.

**Objective:**

We aimed to examine the relationships between people’s perceived severity of the COVID-19 pandemic and their depression, anxiety, and stress to explore the role of cyberchondria, which, in these relationship mechanisms, is closely related to using the internet. In addition, we also examined the moderating role of lockdown experiences.

**Methods:**

In February 2020, a total of 486 participants were recruited through a web-based platform from areas in China with a large number of infections. We used questionnaires to measure participants’ perceived severity of the COVID-19 pandemic, to measure the severity of their cyberchondria, depression, anxiety, and stress symptoms, and to assess their lockdown experiences. Confirmatory factor analysis, exploratory factor analysis, common method bias, descriptive statistical analysis, and correlation analysis were performed, and moderated mediation models were examined.

**Results:**

There was a positive association between perceived severity of the COVID-19 pandemic and depression (β=0.36, *t*=8.51, *P*<.001), anxiety (β=0.41, *t*=9.84, *P*<.001), and stress (β=0.46, *t*=11.45, *P*<.001), which were mediated by cyberchondria (β=0.36, *t*=8.59, *P*<.001). The direct effects of perceived severity of the COVID-19 pandemic on anxiety (β=0.07, *t*=2.01, *P*=.045) and stress (β=0.09, *t*=2.75, *P*=.006) and the indirect effects of cyberchondria on depression (β=0.10, *t*=2.59, *P*=.009) and anxiety (β=0.10, *t*=2.50, *P*=.01) were moderated by lockdown experience.

**Conclusions:**

The higher the perceived severity of the COVID-19 pandemic, the more serious individuals’ symptoms of depression, anxiety, and stress. In addition, the associations were partially mediated by cyberchondria. Individuals with higher perceived severity of the COVID-19 pandemic were more likely to develop cyberchondria, which aggravated individuals’ depression, anxiety, and stress symptoms. Negative lockdown experiences exacerbated the COVID-19 pandemic’s impact on mental health.

## Introduction

### Background

Since 2020, hundreds of millions of people have been infected with COVID-19 and millions of people have died [1]. Due to its long incubation period, high infectiousness, and high risk of death if not treated promptly, COVID-19 has become a major public health emergency worldwide [2]. Public health emergencies, such as severe acute respiratory syndrome (SARS) in 2003 [3], Middle East respiratory syndrome in 2012 [4], and Ebola virus disease in 2014 [5] have significantly harmed people’s lives, caused people to suffer economic losses, and caused severe psychological trauma. The impacts of these events on economic development may be alleviated in the short term, but their impacts on social stability and mental health may be long-term [6]. Studies have shown that, during the COVID-19 pandemic, people experienced varying degrees of depression, anxiety, and stress symptoms [7], which lasted over 4 weeks [8].

Previous studies [9,10] have found that the objective severity of the pandemic is negatively correlated with mental health (eg, depression, anxiety, worry, and dissatisfaction). In addition, knowledge and concerns about COVID-19 (eg, low confidence in doctors, low perceived likelihood of survival, and spending more time gathering health information) [11] and the perceived impact of the pandemic [12] were found to be positively correlated with depression, anxiety, and stress. In this study, we aimed to investigate the relationship between the perceived severity of the COVID-19 pandemic and depression, anxiety, and stress, as well as the mechanisms underlying these associations, with subjective assessments based on psychometric standards.

### Hypothesis 1: Perceived Severity of the COVID-19 Pandemic Is Positively Associated With and Depression, Anxiety, and Stress

The ABC theory of emotions [13] suggests that stimulus events are only indirect causes that trigger individuals’ emotions and behaviors as consequences, while the direct causes of such emotions and behaviors are the beliefs that result from an individual’s perception and evaluation of the stimulus event. One study [14] examined the relationship between individuals’ appraisals of SARS risk and their emotional and behavioral responses. Another study [15] found that the public’s risk perception regarding the Ebola outbreak was positively correlated with fear, anger, anxiety, disgust, and sadness. According to the ABC theory of emotions [13], since the COVID-19 pandemic greatly threatens people’s safety, individuals’ subjective feelings and evaluations of this threat’s severity significantly affect their physical and mental health. Individuals’ mental states may be affected by the pandemic to different degrees depending on their perception of the severity of the COVID-19 pandemic, even while they experience the same event. If individuals perceive the pandemic to be more severe, they are more likely to exhibit negative mental states.

### Hypothesis 2: Cyberchondria Mediates the Association Between Perceived Severity of the COVID-19 Pandemic and Depression, Anxiety, and Stress

With the advent of the digital age, health-related information can be easily and quickly accessed via the internet at little to no cost. Statistics published by the Office for National Statistics [16] show that from 2007 to 2016, the proportion of internet users searching for health-related information increased from 18% to 51%. After the outbreak of COVID-19, people could obtain information on pandemic dynamics on the internet. The unique period of home quarantine also promoted people to use the information found on the internet to diagnose their physical health. During the COVID-19 pandemic, individuals who perceive the pandemic to be more serious are more sensitive to the pandemic’s development and their own health. They repeatedly search for information related to the pandemic to assess their risk of contracting COVID-19. Therefore, the higher individuals’ perceived severity of the COVID-19 pandemic is, the more likely they are to show cyberchondria [17].

Cyberchondria has many negative effects on individuals’ mental health. Research has found that there is a positive correlation between cyberchondria and anxiety during the pandemic [18], and cyberchondria is associated with an increase in searches for health information, which can lead to an individual having irrational thoughts, panicking unnecessarily, and paying excessive attention to health problems and can result in higher levels of depression [19,20]. In addition, after frequent exposure to various types of health-related information, individuals with cyberchondria become even more uncertain about COVID-19 and pay even more attention to their own physical conditions as well as to those of the people around them, which may cause even greater stress. 

### Hypothesis 3: Direct Effects and Indirect Effects Are Moderated by Lockdown Experience

To effectively control the spread of the COVID-19 pandemic and reduce the risk of public infection, many governments adopted public lockdown measures, which included school closures, travel restrictions, and public-gathering bans [21]. These measures effectively controlled the rate and scope of COVID-19 infections by reducing the risk of people becoming infected [22]. However, lockdown policies meant that most communication with the outside world occurred only through telephone or online. This type of social isolation and lack of traditional communication exerts negative psychological effects on people [23,24].

**Figure 1:**
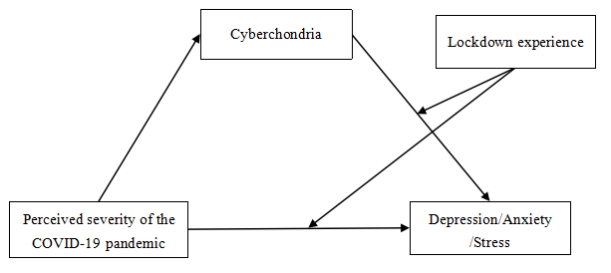
Theoretical model: a moderated mediation model.

Individuals in quarantine may suffer from insomnia and show emotional reactions such as depression, anxiety, stress, anger, and confusion [23,25,26]. In addition, children and adolescents also experienced depression and anxiety during the lockdown—when children and adolescents experienced negative feelings and behaviors during lockdown periods, they were more likely to have symptoms of depression and anxiety, and their mental states were worse than those of children and adolescents without negative lockdown experience [27]. Negative experiences may further aggravate the negative mental state experienced by an individual caused by the COVID-19 pandemic. While individuals with no negative experience are more receptive to lockdown policies and recognize the important role of lockdown measure has in controlling the spread of the pandemic. Thus, the direct effects of perceived severity of the COVID-19 pandemic on depression, anxiety, and stress, and the indirect effects of cyberchondria on depression, anxiety, and stress are moderated by lockdown experience (Figure 1).

## Methods

### Study Design and Participants

From late January to late February 2020, we used a web-based platform to administer questionnaires. A total of 539 participants completed the questionnaires, and 486 participants (137 males and 349 females) were selected, yielding a qualified rate of 90.17%. Participants’ ages ranged from 14 to 50 years (mean 22.94, SD 5.68).The research was approved by the Ethics Committee of the School of Psychology, Shandong Normal University; anonymous testing was used, and the instructions indicated that the data would be used only for scientific research. A small fee was paid to all participants via the internet for their participation.

### Measures

#### Perceived Severity of the COVID-19 Pandemic Questionnaire

We used a self-designed questionnaire to measure the participants’ subjective feelings about the severity of the COVID-19 pandemic. While preparing the questionnaire, we first interviewed 18 people from COVID-19 pandemic areas via web-based videoconference. According to the interview results, perceived severity of the COVID-19 pandemic is divided into 3 dimensions: health risk, emotion, and behavior. Second, we compiled items based on web-based interview results to measure individuals’ perceived severity of the COVID-19 pandemic. Two psychometrics professors were invited to evaluate the questionnaire items and to modify any unclear or ambiguous questions, forming a 26-item preliminary version of the perceived severity of the COVID-19 pandemic questionnaire. Third, 174 participants were recruited and tested using the preliminary questionnaire. The Kaiser-Meyer-Olkin value was 0.83, and Bartlett test of sphericity was significant (*P*<.001), indicating that the data were suitable for factor analysis. Subsequently, we conducted exploratory factor analysis, and items with commonality less than 0.3, factor loadings less than 0.4, and cross-loadings (factor loadings greater than 0.4 in 2 or more dimensions and the difference of factor loadings less than 0.3) were deleted; finally, 14 items remained. Then, confirmatory factor analysis was performed using data from 486 participants. The results showed that the construct validity of the questionnaire was good (model fit index: χ^2^/*df*=3.57, comparative fit index 0.93; Tucker–Lewis index 0.92; root mean square error of approximation 0.07; standardized root mean squared residual 0.06). The questionnaire was based on the 3 dimensions: health risk (eg, “I suspect that anyone may be infected by COVID-19”), emotion (eg, “Because of COVID-19 pandemic, I feel distressed and irritable”), and behavior (eg, “Only after thoroughly disinfecting purchased goods can I use them with peace of mind”). Items were rated on a 5-point scale from 1 (strongly disagree) to 5 (strongly agree); the higher the total score on the questionnaire, the higher perceived severity of the COVID-19 pandemic. Cronbach α=.88, which indicated that the reliability of the questionnaire was good.

#### Lockdown Experience Questionnaire

We used a self-designed questionnaire to measure lockdown experience. While preparing the questionnaire, we first interviewed 16 people from areas in lockdown via web-based videoconference during the COVID-19 pandemic. Based on the interview results, we divided lockdown experience into 3 dimensions: feeling, behavior, and economic situation. Second, we compiled items based on the web-based interview results to measure individuals’ lockdown experiences. Two psychometrics professors were invited to evaluate the questionnaire items and to modify any unclear or ambiguous questions, which formed a 30-item preliminary version of the lockdown experience questionnaire. Third, 174 participants were recruited and tested using the preliminary questionnaire. The Kaiser-Meyer-Olkin value was 0.78, and Bartlett test of sphericity was significant (*P*<.001), indicating that the data were suitable for factor analysis. Subsequently, exploratory factor analysis was carried out, and items with commonality less than 0.3, factor loadings less than 0.4, and cross-loadings were deleted, after which, 11 items remained. Then, confirmatory factor analysis was performed, which included 486 participants. The results showed that the construct validity of the questionnaire was good (model fit index: χ^2^/*df*=2.96, comparative fit index 0.94; Tucker–Lewis index 0.92; root mean square error of approximation 0.06, standardized root mean squared residual 0.05). The questionnaire was based on 3 dimensions: feeling (eg, “During the lockdown period, I feel oppressed”), behavior (eg, “During the lockdown period, my work and learning efficiency decreased”), and economic situation (eg, “I think the lockdown policy has put a lot of pressure on me economically”). Items were rated on a 5-point scale from 1 (strongly disagree) to 5 (strongly agree); higher total scores indicated a more negative lockdown experience. Cronbach α=.77, which indicated that the reliability of the questionnaire is good.

When Liu et al [27] investigated the relationship between lockdown experience and depression and anxiety, they defined and examined the variable of lockdown experience from 2 aspects: feeling and behavior. Based on our interview results, we study divided the dimensions of the lockdown experience into feelings, behavior, and economic situation.

#### Depression Anxiety Stress Scale

Depression, anxiety, and stress were measured using the 21-item Depression Anxiety Stress Scale [28], which is divided into 3 dimensions: depression (eg, “I could see nothing in the future to be hopeful about”), anxiety (eg, “I was worried about situations in which I might panic and make a fool of myself”), and stress (eg, “I found it difficult to relax”). Each dimension contains 7 items, each rated on a 4-point scale from 0 (disagree) to 3 (strongly agree). Higher scores indicate higher levels of depression, anxiety, and stress. During the COVID-19 pandemic, the psychometric properties of the 21-item Depression Anxiety Stress Scale have been verified in samples from different countries [29-38]. In this study, Cronbach α=0.86, 0.85, and 0.90 for the depression, anxiety, and stress subscales, respectively, which indicated that subscale reliability was good.

#### Cyberchondria Scale

To assess cyberchondria, we used the Cyberchondria Scale [39], which is divided into 2 dimensions: impulse and excess (eg, “I spend a lot of time searching for health-related information on the internet”); worry and fear (eg, “When there are different explanations for disease symptoms on the internet, I tend to believe the more serious explanations”). The Cyberchondria Scale consists of 13 items rated on a 4-point scale from 1 (never) to 4 (always), with higher score indicating more serious cyberchondria. Cronbach α=.93, which indicated that the reliability of the scale was good.

### Statistical Analysis

AMOS software (version 7.0; IBM Corp) was used for confirmatory factor analysis. SPSS software (version 24.0; IBM Corp) was used for exploratory factor analysis, common method bias, descriptive statistical analysis, and correlation analysis. SPSS PROCESS macro (version 3.5) was used to verify the moderated mediation models [40]. All regression coefficients were tested using the bias-corrected percentile bootstrap method. The theoretical model was tested by estimating the 95% confidence intervals of the mediation and moderating effects with 5000 repeated samples. An effect was considered significant if the confidence interval did not include 0.

## Results

### Common Method Bias

Because a questionnaire method was used to collect data, which can lead to common method bias, we used the Harman 1-factor test to detect common method bias [41]. The results of principal component factor analysis without rotation showed 14 factors with eigenvalues greater than 1, among which, the variation explained by the first factor was only 26.65%, which is less than the critical standard of 40%. Thus, there was no substantial common method bias in this study.

### Descriptive Statistics and Correlations

We found that perceived severity of the COVID-19 pandemic was positively correlated with depression, anxiety, stress, and cyberchondria and negatively associated with lockdown experience (Table 1). Cyberchondria was positively correlated with depression, anxiety, and stress and negatively associated with lockdown experience. Lockdown experience was negatively associated with depression, anxiety, and stress.

**Table 1:** Means, standard deviations, and correlations among key variables.

Variables		Mean (SD)	Variables					
			Perceived severity	Depression	Anxiety	Stress	Cyberchondria	Lockdown experience
**Perceived severity**		53.66 (8.90)						
	*r*		1	0.36	0.41	0.46	0.36	0.51
	*P* value		—^a^	<.001	<.001	<.001	<.001	<.001
**Depression**		6.68 (7.55)						
	*r*		0.36	1	0.84	0.84	0.38	0.52
	*P* value		<.001	—	<.001	<.001	<.001	<.001
**Anxiety**		6.20 (7.57)						
	*r*		0.41	0.84	1	0.85	0.38	0.50
	*P* value		<.001	<.001	—	<.001	<.001	<.001
**Stress**		9.57 (9.60)						
	*r*		0.46	0.84	0.85	1	0.39	0.54
	*P* value		<.001	<.001	<.001	—	<.001	<.001
**Cyberchondria**		31.64 (8.20)						
	*r*		0.36	0.38	0.38	0.39	1	0.33
	*P* value		<.001	<.001	<.001	<.001	—	<.001
**Lockdown experience**		33.30 (7.06)						
	*r*		0.51	0.52	0.50	0.54	0.33	1
	*P* value		<.001	<.001	<.001	<.001	<.001	—

^a^Not applicable.

### Mediating Effects

In the absence of cyberchondria, the positive predictive effects of perceived severity of the COVID-19 pandemic on depression (β=0.36, *t*=8.51, *P*<.001), anxiety (β=0.41, *t*=9.84, *P*<.001), and stress (β=0.46, *t*=11.45, *P*<.001) were significant (Multimedia Appendix 1). Thus, hypothesis 1 was supported.

When cyberchondria was added to the analysis as a mediator, the direct relationships between perceived severity of the COVID-19 pandemic and depression (β=0.26, *t*=5.85, *P*<.001), anxiety (β=0.31, *t*=7.24, *P*<.001), and stress (β=0.37, *t*=8.83, *P*<.001) were also significant. Perceived severity of the COVID-19 pandemic had a positive predictive effect on cyberchondria (β=0.36, *t*=8.59, *P*<.001). The positive predictive effects of cyberchondria on depression (β=0.29, *t*=6.66, *P*<.001), anxiety (β=0.27, *t*=6.24, *P*<.001), and stress (β=0.26, *t*=6.14, *P*<.001) were also significant.

The results suggested that cyberchondria partially mediated the link between perceived severity of the COVID-19 pandemic and depression (indirect effect 0.11, 95% CI 0.07-0.15). This indirect effect accounted for 30.56% of the total effect. In addition, cyberchondria partially mediated the link between perceived severity of the COVID-19 pandemic and anxiety (indirect effect 0.10, 95% CI 0.06-0.14). This indirect effect accounted for 24.39% of the total effect. Finally, cyberchondria partially mediated the link between perceived severity of the COVID-19 pandemic and anxiety (indirect effect 0.09, 95% CI 0.06-0.14). This indirect effect accounted for 19.57% of the total effect. Thus, hypothesis 2 was supported.

### Moderated Mediation

**Figure 2:**
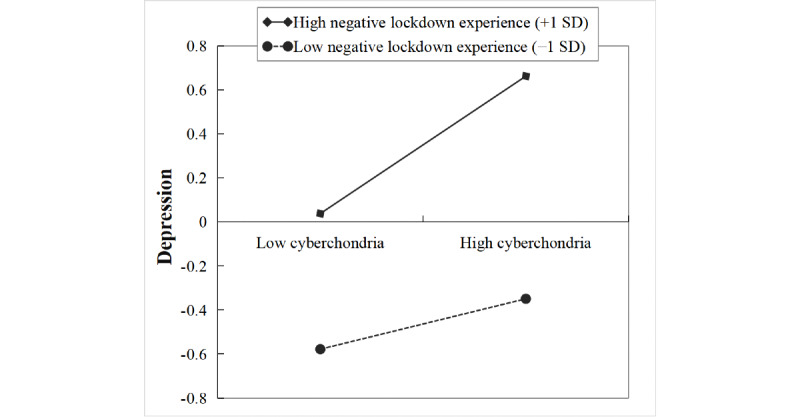
The moderation of the relationship between cyberchondria and depression by lockdown experience.

After lockdown experience (Multimedia Appendix 2) was entered into the model, the product of cyberchondria and lockdown experience had a significant predictive effect on depression (β=0.10, *t*=2.59, *P*=.009), but the product of perceived severity of the COVID-19 pandemic and lockdown experience had no significant predictive effect on depression (β=0.05, *t*=1.38, *P*=.17). Further simple slope analysis (Figure 2) showed that the association between cyberchondria and depression was stronger for individuals with high negative lockdown experience (1 SD above the mean: β=0.31, *t*=5.74, *P*<.001) than that for individuals with low negative lockdown experience (1 SD below the mean: β=0.11, *t*=2.02, *P*=.04).

**Figure 3:**
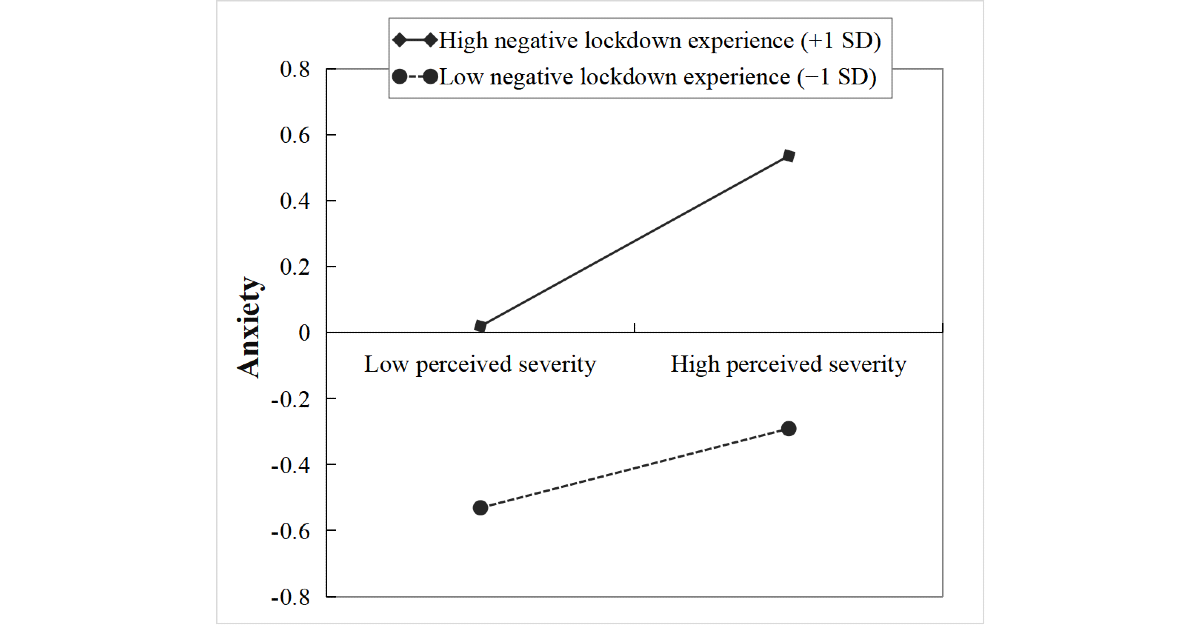
The moderation of the relationship between perceived severity of the COVID-19 pandemic and anxiety by lockdown experience.

The product of perceived severity of the COVID-19 pandemic and lockdown experience (β=0.07, *t*=2.01, *P*=.045) and the product of cyberchondria and lockdown experience (β=0.10, *t*=2.50, *P*=.01) had significant predictive effects on anxiety. Further simple slope analysis (Figure 3) showed that the association between perceived severity of the COVID-19 pandemic and anxiety was stronger for individuals with high negative lockdown experience (1 SD above the mean: β=0.26, *t*=4.15, *P*<.001) than that for individuals with low negative lockdown experience (1 SD below the mean: β=0.12, *t*=2.33, *P*=.02).

**Figure 4:**
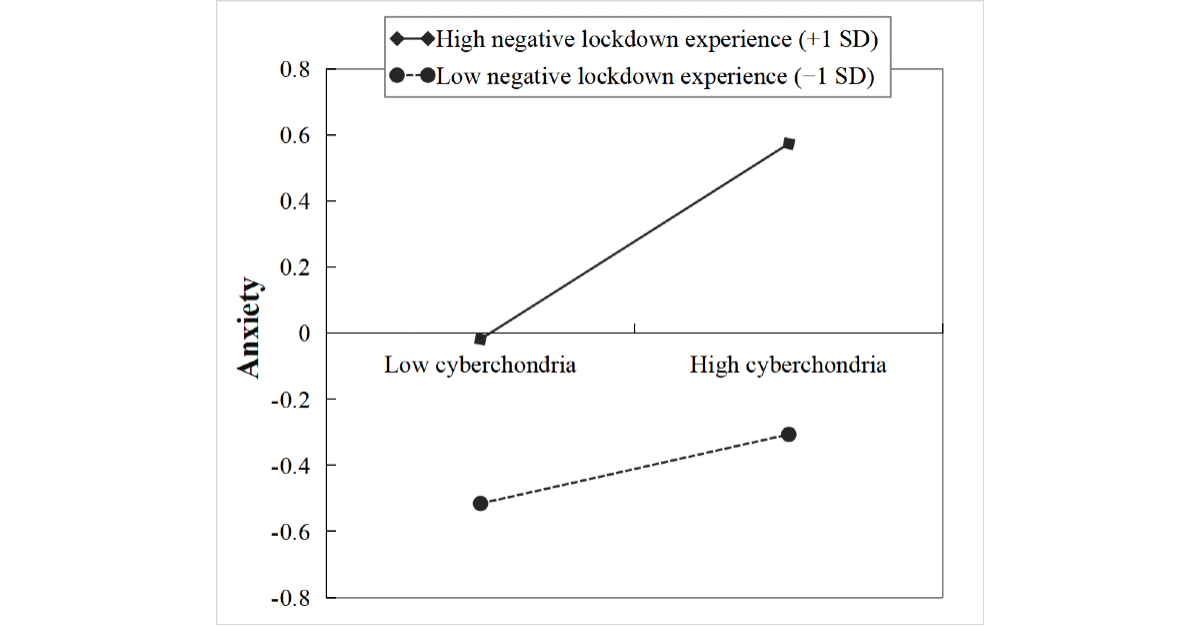
The moderation of the relationship between cyberchondria and anxiety by lockdown experience.

Similarly, simple slope analysis (Figure 4) indicated that the association between cyberchondria and anxiety was stronger for individuals with high negative lockdown experiences (1 SD above the mean: β=0.30, *t*=5.43, *P*<.001) than that for individuals with low negative lockdown experience (1 SD below the mean: β=0.10, *t*=1.84, *P*=.07).

**Figure 5:**
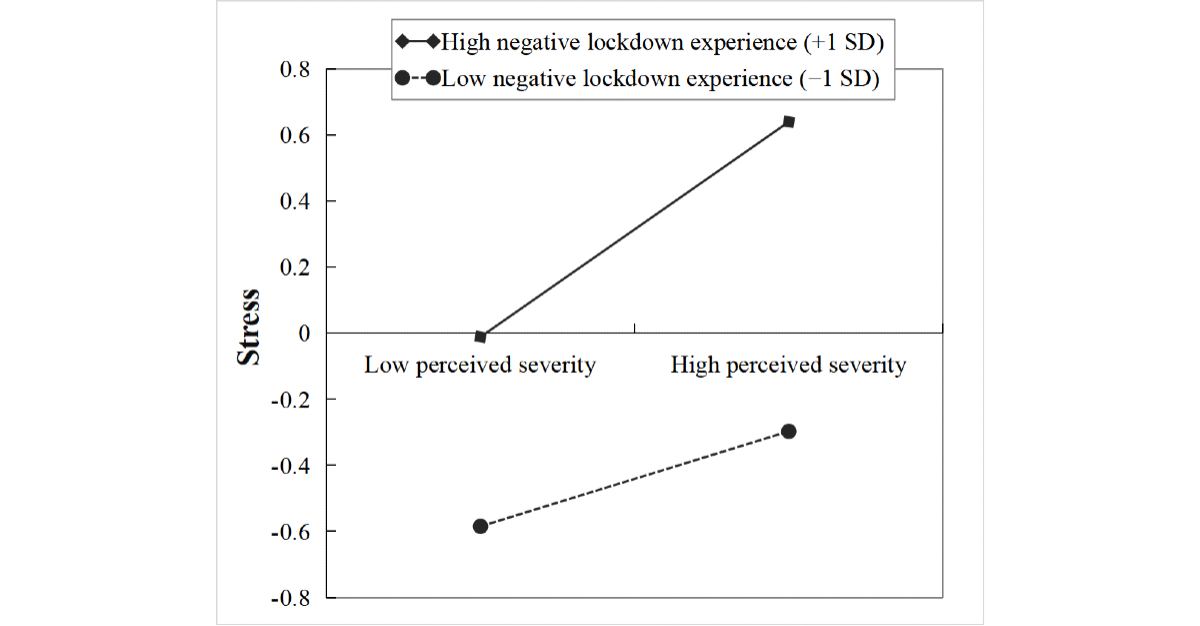
The moderation of the relationship between perceived severity of the COVID-19 pandemic and stress by lockdown experience.

The product of perceived severity of the COVID-19 pandemic and lockdown experience had a significant predictive effect on stress (β=0.09, *t*=2.75, *P*=.006), but the product of cyberchondria and lockdown experience had no significant predictive effect on stress (β=0.05, *t*=1.44, *P*=.15). Further simple slope analysis (Figure 5) showed that the association between perceived severity of the COVID-19 pandemic and stress was stronger for individuals with high negative lockdown experience (1 SD above the mean: β=0.33, *t*=5.46, *P*<.001) than that for individuals with low negative lockdown experience (1 SD below the mean: β=0.14, *t*=2.91, *P*=.004).

These results indicated that individuals’ higher negative lockdown experience strengthened the positive effect of perceived severity of the COVID-19 pandemic on anxiety and stress and the positive effect of cyberchondria on depression and anxiety. Thus, hypothesis 3 was partially supported.

## Discussion

### Principal Findings

In this study, we found that perceived severity of the COVID-19 pandemic was positively associated with depression, anxiety, and stress. The higher individuals’ perceived severity of the COVID-19 pandemic, the higher their levels of depression, anxiety, and stress. The severity of cyberchondria partly mediated the relationship between perceived severity of the COVID-19 pandemic and depression, anxiety, and stress. Individuals with high perceived severity of the COVID-19 pandemic were more likely to suffer from cyberchondria, and the higher the severity of cyberchondria, the higher their depression, anxiety, and stress levels. The direct effect of perceived severity of the COVID-19 pandemic on anxiety and stress and the indirect effect of cyberchondria on depression and anxiety were moderated by the lockdown experience. Individuals with high negative lockdown experience had stronger relationships between perceived severity of the COVID-19 pandemic and anxiety/stress and between cyberchondria and depression/anxiety.

### Perceived Severity of the COVID-19 Pandemic and Depression, Anxiety, and Stress

Perceived severity of the COVID-19 pandemic had a significant positive predictive effect on depression (*P*<.001), anxiety (*P*<.001), and stress (*P*<.001), which is consistent with the findings of previous studies [9,10] on the objective severity of the COVID-19 pandemic and supports the ABC theory of emotions [13]. This finding indicates that the COVID-19 pandemic has prompted a series of emotional reactions that increase with perceived severity of the COVID-19 pandemic. When individuals thought that the pandemic was very serious and were not able to deal with it well, the negative impact of the pandemic increased.

Specifically, in the COVID-19 public health emergency, individuals with higher perceived severity of the COVID-19 pandemic perceived a greater threat to their safety; therefore, they were worried and panicked about the spread of the pandemic for an extended time period, which increased their depression, anxiety, and stress levels. In contrast, individuals with lower perceived severity of the COVID-19 pandemic thought that the spread of the pandemic could be effectively controlled; therefore, they did not worry too much about their safety, which allowed their depression, anxiety, and stress levels to be lower.

### The Mediating Role of Cyberchondria

Cyberchondria moderated the association between perceived severity of the COVID-19 pandemic and depression (*P*<.001), anxiety (*P*<.001), and stress (*P*<.001), which is consistent with previous findings. Laato et al [42] found that individuals’ cyberchondria worsened as individuals’ perceived severity of the COVID-19 pandemic increased. According to the ABC theory of emotions [13], individuals with higher perceived severity of the COVID-19 pandemic would continue to pay attention to the pandemic and believe that they were at high risk of contracting COVID-19, and they would repeatedly search for information related to the COVID-19 pandemic. Moreover, excessive or repetitive internet searches for health-related information are one of the main causes of cyberchondria. Many people’s concerns about illness are not alleviated by searching for related information, but instead, are further aggravated [43]. Therefore, individuals with higher perceived severity of the COVID-19 pandemic have a higher degree of cyberchondria than individuals with lower perceived severity of the COVID-19 pandemic.

In this study, we found that individuals with higher perceived severity of the COVID-19 pandemic had higher levels of depression, anxiety, and stress when they showed higher levels of cyberchondria. Consistent with the findings of previous studies [44,45], individuals searching the internet for health-related information did not reduce their concerns about illness but rather increased their levels of depression and anxiety. We further explored the relationship between cyberchondria and stress. The results support the hypothesis that one’s stress level is higher when one’s cyberchondria is more severe. Specifically, when individuals were worried about their illness, they searched for health-related information to eliminate their worries. However, individuals with severe cyberchondria often think that the reliability of health-related information obtained via internet search is very low, and they still worry about their illness after the search [46,47]. During the outbreak of the COVID-19 pandemic, the internet searching behavior of individuals with higher severity of cyberchondria continued for an extended amount of time. Their chronic negative state of fear that they were infected with COVID-19 increased their levels of depression, anxiety, and stress.

### The Moderating Role of Lockdown Experience

We found that lockdown experience moderated the direct effects of perceived severity of the COVID-19 pandemic on anxiety (*P*=.045) and stress (*P*=.006). Our findings are consistent with those of a previous study [27] that showed that lockdown measures are usually associated with a negative mental state. During lockdown, people remained in their homes for an extended period of time, had to abandon their daily routines, and rarely had social contact with others, which caused them to suffer from feelings of boredom, frustration, and isolation [23]. Individuals who were affected by lockdown measures may have experienced life problems and had more serious negative experiences for example, they may have believed that the lockdown measures affected their quality of life and economic resources, which aggravated their anxiety and stress caused by their perceived severity of the COVID-19 pandemic. In contrast, for individuals with a less negative lockdown experience, this measure did not affect them as negatively, and they were more likely to recognize the important role of lockdown measures in controlling the COVID-19 pandemic. Therefore, the anxiety and stress caused by perceived severity of the COVID-19 pandemic could be alleviated.

The findings of our study also suggested that lockdown experience moderated the negative effects of cyberchondria on depression (*P*=.009) and anxiety (*P*=.01). During the COVID-19 pandemic, everyone was subject to the lockdown policy, but compared to individuals with a high degree of negative lockdown experience, individuals with a low degree of negative lockdown experience usually thought that the lockdown policy implemented by the government could effectively control the spread of the pandemic and help reduce the likelihood that they would be infected with COVID-19. Therefore, a low degree of negative lockdown experience could reduce the depression and anxiety caused by cyberchondria.

### Implications and Limitations

The public should be guided to calmly seek pandemic-related knowledge, to prevent a series of negative emotional reactions. Countries and governments should also promptly control the spread of the COVID-19 pandemic and curb the spread of false or exaggerated information related to the pandemic, which will help alleviate cyberchondria and reduce depression, anxiety, and stress levels. Simultaneously, lockdown experiences’ impact on individuals’ psychological states should also be considered. Therefore, in implementing a lockdown policy, the government should reduce the public’s degree of negative lockdown experience as much as possible by issuing unemployment benefits and wage subsidies and providing accommodations. These approaches can help the government control the COVID-19 pandemic and alleviate people’s negative mental states and psychological problems due to the outbreak of the pandemic. In addition, previous studies have shown that the most evidence-based treatment for psychiatric symptoms during COVID-19 is cognitive behavioral therapy [48]. In particular, internet cognitive behavioral therapy can effectively treat individuals’ symptoms of depression, anxiety, and cyberchondria and can also reduce insomnia [49-51]. Therefore, internet cognitive behavioral therapy can be used to treat people’s psychiatric symptoms during the COVID-19 pandemic, which can provide people with convenient, fast, and effective psychological assistance during the lockdown period [52].

This study also had several limitations. The COVID-19 pandemic was found to cause hemodynamic changes in the brain [53]. This study mainly used self-reported questionnaires to measure psychiatric symptoms and did not make a clinical diagnosis. The gold standard for establishing psychiatric diagnosis involves a structured clinical interview and functional neuroimaging [54-56]. In the future, more technical means, combined with clinical diagnostic criteria, must be adopted to investigate the impact of major public health emergencies (such as the COVID-19 pandemic) on mental health. In addition, this study was cross-sectional in design and could not identify causal relationships among the variables. Moreover, data were collected during the high-incidence stage of China’s pandemic, which means that the results reflect only the mental health status of the Chinese public during this stage of the disease but do not reveal the dynamic changes in the relationships between the variables. A longitudinal study should be used to explore the COVID-19 pandemic’s continuous impact on people’s psychology.

### Conclusions

This study showed that the higher individuals’ perceived severity of the COVID-19 pandemic was, the higher their levels of depression, anxiety, and stress. Cyberchondria partially mediated the relationships between perceived severity of the COVID-19 pandemic and depression, anxiety, and stress. Individuals with higher perceived severity of the COVID-19 pandemic were more likely to develop cyberchondria and had higher depression, anxiety and stress levels. The lockdown experience moderated the direct effect of perceived severity of the COVID-19 pandemic on anxiety/stress and the indirect effects of cyberchondria on depression/anxiety. A high degree of negative lockdown experience could exacerbate the negative influence of perceived severity of the COVID-19 pandemic on anxiety/stress as well as the negative influence of cyberchondria on depression/anxiety.

## Appendix

Multimedia Appendix 1 Mediating effect analysis.

Multimedia Appendix 2 Mediating effect analysis with moderation.
