# Supplementary material for: Associations Between the Perceived Severity of the COVID-19 Pandemic, Cyberchondria, Depression, Anxiety, Stress, and Lockdown Experience: Cross-sectional Survey Study
Source: JMIR Public Health Surveill. 2021 Sep 16;7(9):e31052. doi: 10.2196/31052 (PMC8448083; doi:10.2196/31052)
Supplement: Multimedia Appendix 1 [file publichealth_v7i9e31052_app1.doc]

**Multimedia Appendix 1. Mediating effect analysis.**

| Regression equation  Outcome variables | Goodness of fit  Predictive variables | Coefficient significance  *R^2^* | *F* value | *β* value | *t* value | *P* value |
| --- | --- | --- | --- | --- | --- | --- |
| **Depression** |  | 0.13 | 72.46 |  |  | <.001 |
|  | Perceived severity |  |  | 0.36 | 8.51 | <.001 |
| **Anxiety** |  | 0.17 | 96.74 |  |  | <.001 |
|  | Perceived severity |  |  | 0.41 | 9.84 | <.001 |
| **Stress** |  | 0.21 | 131.18 |  |  | <.001 |
|  | Perceived severity |  |  | 0.46 | 11.45 | <.001 |
| **Cyberchondria** |  | 0.13 | 73.86 |  |  | <.001 |
|  | Perceived severity |  |  | 0.36 | 8.59 | <.001 |
| **Depression** |  | 0.20 | 61.69 |  |  | <.001 |
|  | Perceived severity |  |  | 0.26 | 5.85 | <.001 |
|  | Cyberchondria |  |  | 0.29 | 6.66 | <.001 |
| **Anxiety** |  | 0.23 | 71.62 |  |  | <.001 |
|  | Perceived severity |  |  | 0.31 | 7.24 | <.001 |
|  | Cyberchondria |  |  | 0.27 | 6.24 | <.001 |
| **Stress** |  | 0.27 | 89.40 |  |  | <.001 |
|  | Perceived severity |  |  | 0.37 | 8.83 | <.001 |
|  | Cyberchondria |  |  | 0.26 | 6.14 | <.001 |
