# Supplementary material for: Associations Between the Perceived Severity of the COVID-19 Pandemic, Cyberchondria, Depression, Anxiety, Stress, and Lockdown Experience: Cross-sectional Survey Study
Source: JMIR Public Health Surveill. 2021 Sep 16;7(9):e31052. doi: 10.2196/31052 (PMC8448083; doi:10.2196/31052)
Supplement: Multimedia Appendix 2 [file publichealth_v7i9e31052_app2.doc]

**Multimedia Appendix 2.** Mediating effect analysis with moderation.

| Regression equation  Outcome variables | Goodness of fit  Predictive variables | Coefficient significance  *R^2^* | *F* value | *β* value | *t* value | *P value* |
| --- | --- | --- | --- | --- | --- | --- |
| **Depression** |  | 0.34 | 49.83 |  |  | <.001 |
|  | Perceived severity |  |  | 0.10 | 2.18 | =.03 |
|  | Cyberchondria |  |  | 0.21 | 5.27 | <.001 |
|  | Lockdown experience |  |  | 0.41 | 9.30 | <.001 |
|  | Perceived severity × Lockdown experience |  |  | 0.05 | 1.38 | =.17 |
|  | Cyberchondria × Lockdown experience |  |  | 0.10 | 2.59 | =.009 |
| **Anxiety** |  | 0.34 | 48.78 |  |  | <.001 |
|  | Perceived severity |  |  | 0.19 | 4.16 | <.001 |
|  | Cyberchondria |  |  | 0.20 | 4.93 | <.001 |
|  | Lockdown experience |  |  | 0.34 | 7.84 | <.001 |
|  | Perceived severity × Lockdown experience |  |  | 0.07 | 2.01 | =.045 |
|  | Cyberchondria × Lockdown experience |  |  | 0.10 | 2.50 | =.01 |
| **Stress** |  | 0.39 | 61.48 |  |  | <.001 |
|  | Perceived severity |  |  | 0.23 | 5.38 | <.001 |
|  | Cyberchondria |  |  | 0.19 | 4.76 | <.001 |
|  | Lockdown experience |  |  | 0.38 | 8.96 | <.001 |
|  | Perceived severity × Lockdown experience |  |  | 0.09 | 2.75 | =.006 |
|  | Cyberchondria × Lockdown experience |  |  | 0.05 | 1.44 | =.15 |
